# Engineering of *Saccharomyces cerevisiae* for anthranilate and methyl anthranilate production

**DOI:** 10.1186/s12934-021-01532-3

**Published:** 2021-02-03

**Authors:** Joosu Kuivanen, Matti Kannisto, Dominik Mojzita, Heiko Rischer, Mervi Toivari, Jussi Jäntti

**Affiliations:** 1 VTT Technical Research Centre of Finland Ltd, Espoo, Finland; 2 Present Address: eniferBio Oy, Espoo, Finland

**Keywords:** Anthranilate, Methyl anthranilate, *Saccharomyces cerevisiae*, Metabolic engineering, Shikimate pathway

## Abstract

**Background:**

Anthranilate is a platform chemical used by the industry in the synthesis of a broad range of high-value products, such as dyes, perfumes and pharmaceutical compounds. Currently anthranilate is produced via chemical synthesis from non-renewable resources. Biological synthesis would allow the use of renewable carbon sources and avoid accumulation of toxic by-products. Microorganisms produce anthranilate as an intermediate in the tryptophan biosynthetic pathway. Several prokaryotic microorganisms have been engineered to overproduce anthranilate but attempts to engineer eukaryotic microorganisms for anthranilate production are scarce.

**Results:**

We subjected *Saccharomyces cerevisiae*, a widely used eukaryotic production host organism, to metabolic engineering for anthranilate production. A single gene knockout was sufficient to trigger anthranilate accumulation both in minimal and SCD media and the titer could be further improved by subsequent genomic alterations. The effects of the modifications on anthranilate production depended heavily on the growth medium used. By growing an engineered strain in SCD medium an anthranilate titer of 567.9 mg l^−1^ was obtained, which is the highest reported with an eukaryotic microorganism. Furthermore, the anthranilate biosynthetic pathway was extended by expression of anthranilic acid methyltransferase 1 from *Medicago truncatula*. When cultivated in YPD medium, this pathway extension enabled production of the grape flavor compound methyl anthranilate in *S. cerevisiae* at 414 mg l^−1^.

**Conclusions:**

In this study we have engineered metabolism of *S. cerevisiae* for improved anthranilate production. The resulting strains may serve as a basis for development of efficient production host organisms for anthranilate-derived compounds. In order to demonstrate suitability of the engineered *S. cerevisiae* strains for production of such compounds, we successfully extended the anthranilate biosynthesis pathway to synthesis of methyl anthranilate.

## Background

Aromatic compounds varying from platform chemicals, such as polymer precursors, to high-value fine chemicals including flavour, fragrance and pharmaceutical substances are widely used in different industries. These compounds are typically derived from benzene, toluene and xylene obtained from petroleum or natural gas, or from secondary metabolites extracted from plant tissues. Recently, renewable production of aromatics and their derivatives using microbes has gained an increasing interest [1–4]. Among the compounds that are currently derived from petroleum, but could also be produced from renewables using industrial biotechnology, is the aromatic amine anthranilate (ANTH). ANTH is used for example as a precursor molecule in the production of dyes, fragrances and pharmaceuticals [5, 6]. Its derivative methyl anthranilate (Me-ANTH), a naturally occurring plant-derived compound [7], constitutes a food flavour and cosmetic ingredient of significant demand.

ANTH is an intermediate in the microbial tryptophan (Trp) biosynthetic pathway and its production has been reported using engineered *Escherichia coli* [8], *Pseudomonas putida* [5] and *Corynebacterium glutamicum* [9] cells. In addition, production of a polymer precursor cis,cis-muconic acid (CCM) via ANTH in *E. coli* cells [10], and the flavor compound Me-ANTH in *E. coli* and *C. glutamicum* [9] have been reported.

Besides bacterial production hosts, the yeast *Saccharomyces cerevisiae* has raised interest as a chassis for aromatics production due to its robustness in bioprocess conditions [11] and the capability to express eukaryotic cytochrome P450 oxidases [12]–key enzymes in aromatic secondary metabolite pathways. *S. cerevisiae* has been engineered to convert ANTH to cinnamoyl, dihydrocinnamoyl and benzoyl anthranilates, potential pharmaceuticals [13]. These compounds were not however produced de novo*,* but ANTH feeding was required. Hitherto, ANTH production in *S. cerevisiae* had only been outlined in the patent literature [14]. Development and investigation of a *S. cerevisiae* platform for de novo ANTH production could provide a platform for ANTH and its many derivatives, and give useful insight of the less frequently engineered Trp pathway in *S. cerevisiae*.

**Fig. 1:**
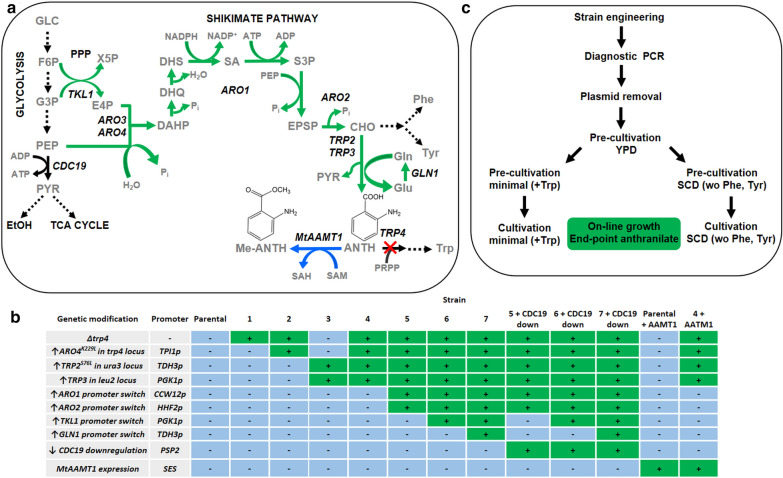
**a** A simplified representation of the metabolic pathways engineered in this study. The upregulated reactions are shown with green arrows and the eliminated reaction is shown with a crossed arrow. The reaction added to extend the pathway to Me-ANTH synthesis is shown with a blue arrow. **b** Modifications done to the strains generated in this study. **c** Workflow of the construction and characterization of the strains

Similarly to bacterial hosts, aromatic compounds in *S. cerevisiae* are mainly synthesized through the shikimate (SA) pathway and the following aromatic amino acid pathways for phenylalanine (Phe), tyrosine (Tyr) and Trp (Fig. 1a). SA pathway begins with the aldol reaction of erythrose-4-phosphate (E4P) and phosphoenolpyruvate (PEP) forming 3-deoxy-D-arabino-heptulosonate-7-phosphate (DAHP), catalysed by the DAHP synthases (EC 2.5.1.54) Aro3 and Aro4. In the next five reactions, DAHP is converted to 5-enolpyruvyl-shikimate-3-phosphate (EPSP) via 3-dehydroquinate (DHQ), 3-dehydroshikimate (DHS), SA and shikimate-3-phosphate (S3P). In bacteria, these reactions are catalysed by separate enzymes but in *S. cerevisiae*, the conversion is carried out by the action of the pentafunctional enzyme Aro1 [15]. The last reaction from EPSP is catalysed by Aro2 resulting in formation of chorismate (CHO)–a common precursor of the aromatic amino acids. After CHO formation, separate branches diverge for the biosynthesis of Phe, Tyr and Trp. ANTH is the first intermediate in the Trp biosynthetic pathway and it is produced by Trp2/Trp3 complex in a reaction where an amide group is transferred from glutamine (Gln) to CHO resulting in formation of ANTH and glutamate (Glu). Gln synthase encoded by *GLN1* regenerates the consumed Gln from Glu and ammonia. The SA pathway is regulated by the allosteric inhibition of several enzymes, such as, Aro3, Aro4 [4] and Trp2. In addition, the flux through the SA pathway is limited by the availability of PEP, which is converted to pyruvate by the main pyruvate kinase of *S. cerevisiae*, encoded by *CDC19* [16]. The availability of E4P from the pentose phosphate pathway (PPP) can also be considered a limiting factor because the carbon flux through E4P is low in *S. cerevisiae* [17].

In this study, the de novo production of ANTH is investigated in *S. cerevisiae*. Several upstream pathway genes were overexpressed by editing the chromosomal promoter regions of the target genes using a rapid CRISPR/Cas9 genome editing approach. In addition, the allosteric regulation of the SA pathway and the Trp branch was relieved with chromosomally integrated mutant enzymes. One of the ANTH overproduction strains was further engineered to produce the grape flavour compound, Me-ANTH, by expressing anthranilic acid methyltransferase 1 (MtAAMT1) from *Medicago truncatula*. To the best of our knowledge, besides the patent literature [14] describing ANTH production in *S. cerevisiae*, this is the first report on engineering an eukaryotic strain for ANTH or Me-ANTH production.

## Results

### Construction and characterization of the strains

We used a prototroph *S. cerevisiae* strain CEN.PK113-1A as a platform for the engineering of the ANTH-producing *S. cerevisiae* strain. Gene deletions and the replacements of the native target gene promoters were generated using the two-plasmid CRISPR/Cas9 system described previously [18]. The plasmid expressing an sgRNA was transformed as PCR amplified fragments into a target *S. cerevisiae* strain that already maintained the Cas9 expression plasmid (see Additional file 1: Fig. S1), a method shown to enable a quick turnaround time in each engineering cycle [19]. The strains (Fig. 1b) were cultivated in microtiter plates using a cultivation robot [18], and both the optical density (OD) values together with the ANTH production were measured at the end of cultivation. Two different cultivation media were tested–the SCD medium containing 2% glucose and amino acids (except Phe and Tyr) and the Verduyn minimal medium containing 1% glucose and supplemented by Trp (due to the auxotrophy after the absence of Trp4 activity). The modified metabolic steps, generated strain and workflow are presented in Fig. 1a–c, respectively.

### Triggering anthranilate accumulation by disruption of *TRP4*

**Fig. 2:**
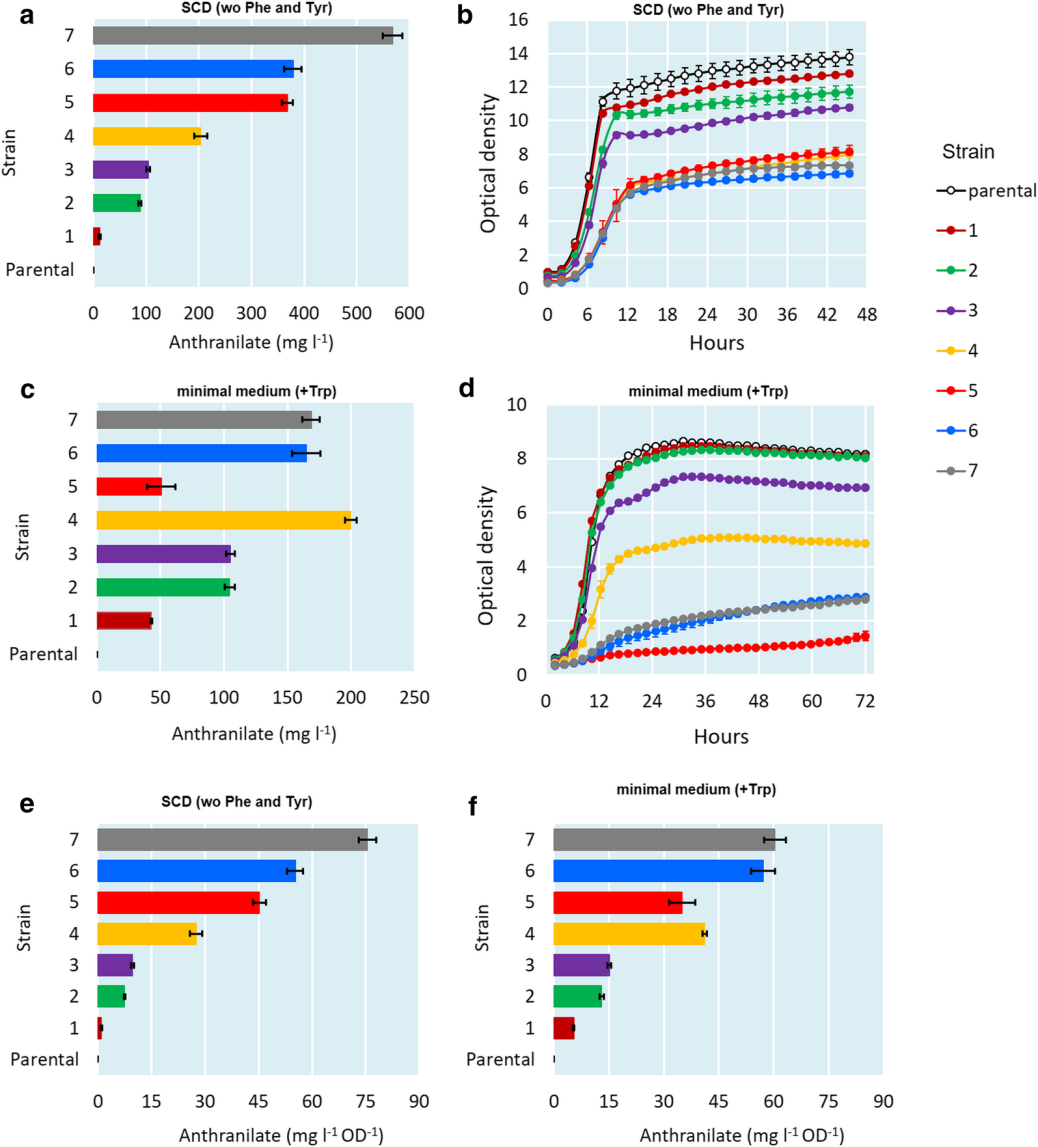
ANTH titers at the end-point of the cultivations of the engineered strains 1–7 in SCD (**a**) and minimal medium (**c**). ODs of the cultivations in SCD (**b**) and minimal medium (**d**). The end-point ANTH titers divided by the end-point ODs of the engineered strains 1–7 in SCD (**e**) and minimal medium (**f**)

The wild type strain did not produce ANTH in either media. When the *TRP4* gene, encoding an ANTH phosphoribosyl transferase, was disrupted (strain 1), ANTH titers of 11.4 and 42.9 mg l^−1^ were detected in SCD and minimal medium, respectively (Fig. 2a, c). A slight growth defect caused by *TRP4*-deletion was observed in SCD but not in minimal medium (Fig. 2b, d).

### Relieving allosteric regulation of Aro4 and Trp2 and overexpression of *TRP3*

To overcome feedback-inhibition of the DAHP synthases, we introduced the feedback insensitive variant *ARO4*^*K229L*^ [20] of a DAHP synthase as a single-copy genomic integration into the *trp4* locus without removing the native *ARO4* (strain 2). This modification improved ANTH titer in SCD medium to 88.4 mg l^−1^ and in minimal medium to 104.5 mg l^−1^, but caused a small growth defect in SCD medium (Fig. 2b).

A feedback insensitive mutant *TRP2*^*S76L*^ [21] was introduced together with *TRP3* overexpression cassette as single-copy genomic integrations into the *ura3* and *leu2* loci, respectively, of the parental strain (to form strain 3) and the *ΔTRP4*-*ARO4*^*K229L*^ strain (to form strain 4) (Fig. 1b). Interestingly, the strain 3 without the deletion of *TRP4* nor the expression of *ARO4*^*K229L*^ produced similar titers of ANTH and had a more severe growth defect compared to strain 2. It is unlikely for the growth defects in the ANTH-producing strains described here to be due to toxicity of ANTH since it has been shown that yeast growth is not inhibited even at an ANTH concentration of 2 g l^−1^ [22]. When both feedback insensitive enzymes Aro4^K229L^ and Trp2^S76L^ were combined (strain 4), the observed ANTH titers were doubled in both SCD (209.9 mg l^−1^) and minimal (199.7 mg l^−1^) media. Also the growth reduced significantly compared to the previous strains 1–3 (Fig. 2b, d).

### Activation of *ARO1*, *ARO2* and *TKL1* by promoter replacements

Next, the SA pathway genes *ARO1* and *ARO2* were overexpressed by replacing the native promoters with known strong constitutive yeast promoters (Fig. 1b). The replacement was implemented through duplexed CRISPR genome edits into strain 4, forming strain 5. In SCD medium, the observed ANTH titer increased from 209.9 mg l^−1^ (strain 4) up to 367.9 mg l^−1^ while the growth remained at the same level (Fig. 2a, b). In contrast, a severe growth defect was observed in minimal medium resulting in low ANTH titer (Fig. 2c, d). However, when ANTH titer was normalized to OD (Fig. 2e, f), the production in minimal medium by the strain 5 (35.0 mg l^−1^ OD^−1^) remained at a higher level than with the strains 1–3 (5.3–15.2 mg l^−1^ OD^−1^), but still lower as compared to the strain 4 (41.1 mg l^−1^ OD^−1^).

Overexpression of the transketolase *TKL1* potentially increasing flux towards E4P into strain 5 (resulting in strain 6) had a slightly negative effect on growth as compared to strain 5, but no significant effect on ANTH titer in SCD (Fig. 2a, b). However, OD normalized ANTH production was increased from 45.2 mg l^−1^ OD^−1^ to 55.2 mg l^−1^ OD^−1^ (Fig. 2e). In minimal medium, the growth defect resulting from *ARO1* and *ARO2* overexpression in strain 5 was partially restored (Fig. 2d). In addition, the OD normalized ANTH production was higher (57.1 mg l^−1^ OD^−1^) compared to any of the previous strains 1–5 (5.3–41.1 mg l^−1^ OD^−1^) (Fig. 2f).

### Overexpression of the Gln synthase *GLN1*

To ensure sufficient Gln supply, we activated the native *GLN1* expression by replacing its native promoter with a strong constitutive yeast promoter (Fig. 1b). As a result, when grown in SCD medium, we observed a significant increase in the ANTH titer from 378.8 mg l^−1^ (strain 6) to 567.9 mg l^−1^ (strain 7) (Fig. 2a). At the same time, only a slight reduction on growth was observed (Fig. 2b). However, the benefit from *GLN1* activation was not observed when the strain was grown in minimal medium (Fig. 2c, d, f).

### Down-regulating the pyruvate kinase *CDC19*

**Fig. 3:**
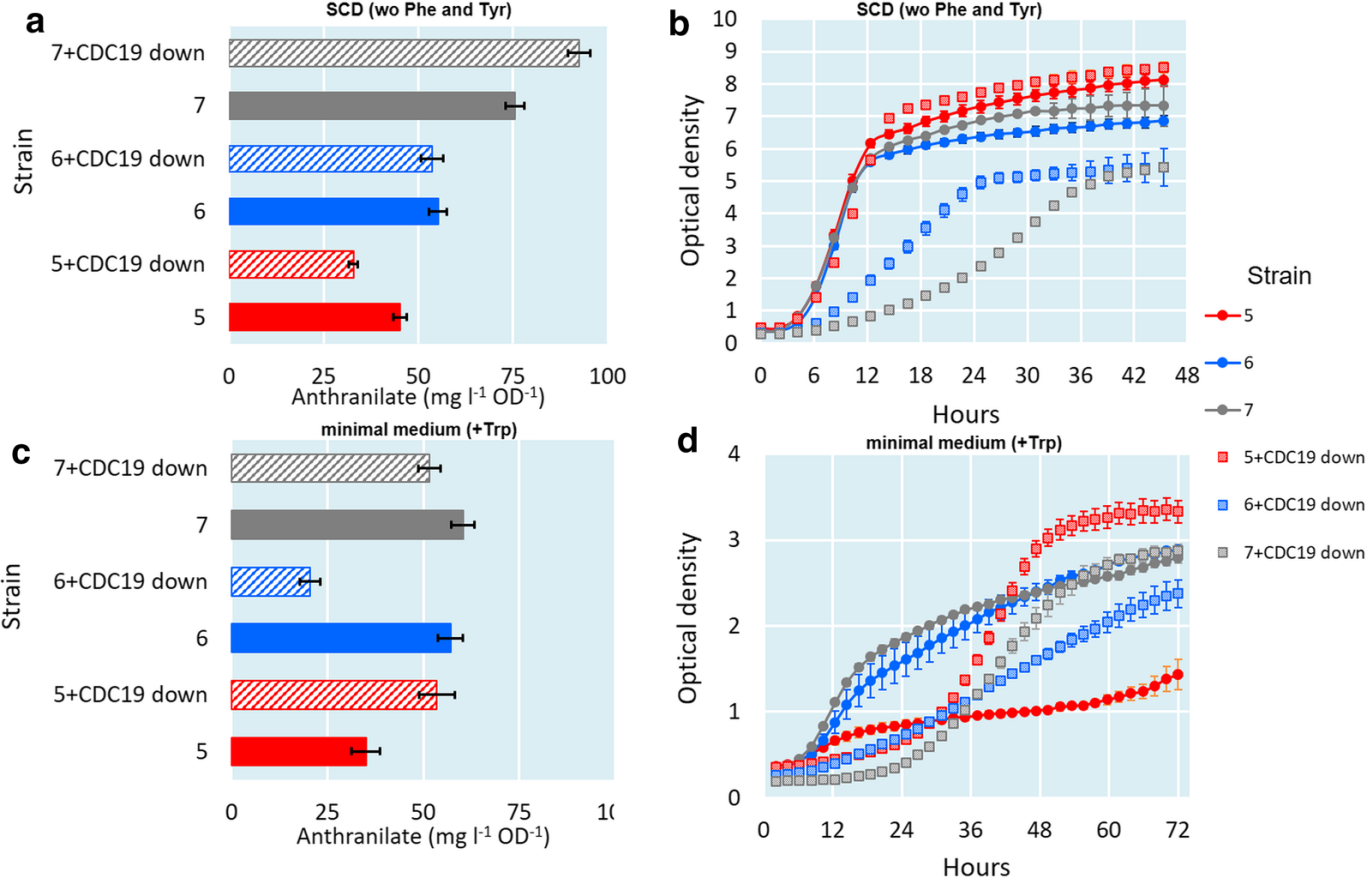
ANTH titers at the end-point of the cultivations of the strains 5, 6 and 7 with and without *CDC19* downregulation in SCD (**a**) and minimal medium (**c**). ODs of the cultivations in SCD (**b**) and minimal medium (**d**)

We further attempted to increase the PEP flux towards ANTH by regulating the *CDC19* expression in the strains 5, 6 and 7. Attempts to generate a glucose-growing null mutant failed, but we succeeded in replacing the native promoter with the *PSP2* promoter that has been shown to be a weak promoter [23]. In SCD medium, the OD normalized ANTH production improved slightly in the modified strain 7 (from 75.5 to 92.4 mg l^−1^ OD^−1^) but not in the modified strains 5 (from 45.2 to 32.8 mg l^−1^ OD^−1^) and 6 (from 55.2 to 53.6 mg l^−1^ OD^−1^) (Fig. 3a). A clear growth defect was observed in the modified strains 6 and 7, but not in the strain 5. In contrast, only the strain 5 showed an increase in the ANTH titer (from 35.0 to 53.5 mg l^−1^ OD^−1^) in minimal medium (Fig. 3c). However, the growth of the strain 5 without the *CDC19* down-regulation (Fig. 3d) was very poor, which may affect this observation. Interestingly, the *CDC19* down-regulation restored the growth of strain 5 significantly. With the strains 6 and 7 in minimal medium, the downregulation affected the growth negatively.

### Production of methyl anthranilate by expression of the *MtAAMT1* gene

**Fig. 4:**
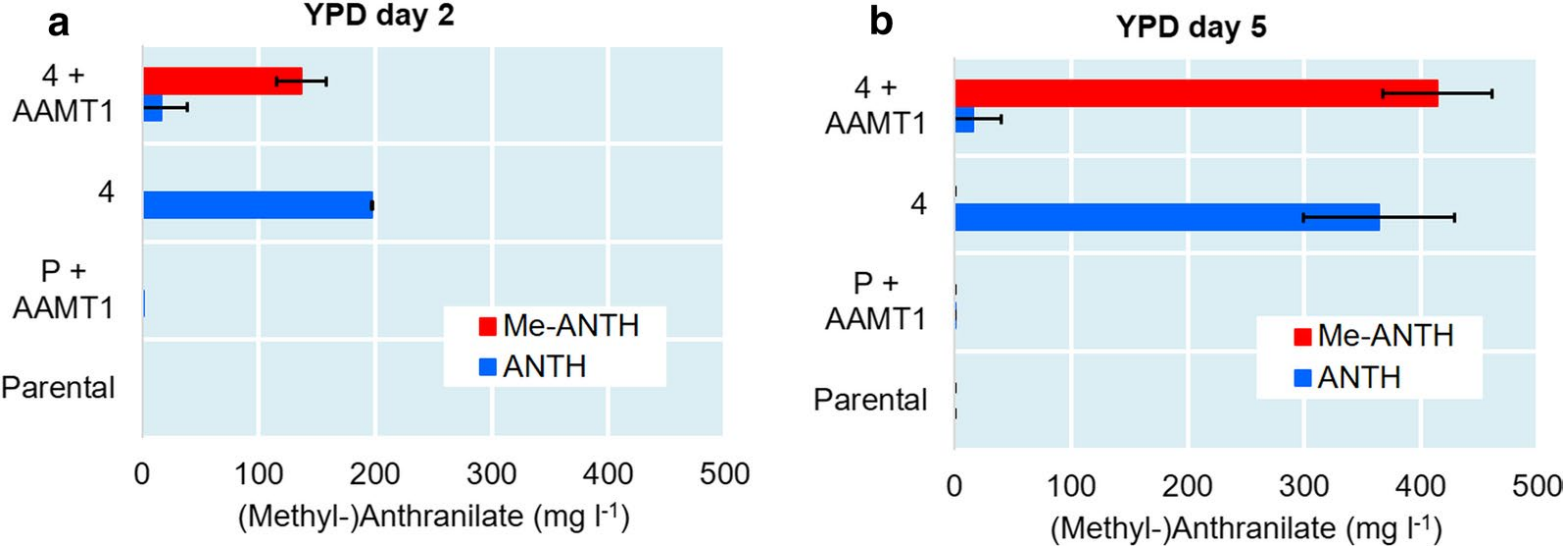
ANTH and Me-ANTH titers in the cultivations of the parental strain and the strain 4, with and without the *MtAAMT1* expression cassette. The cultivations were performed in YPD medium and the products analysed in the culture supernatants at day 2 (**a**) and day 5 (**b**)

The strain 4 was selected a base strain for the production of Me-ANTH due to its relatively fast growth and yet decent production of ANTH as compared to more engineered strains observed in minimal medium (Fig. 2c, d). The *MtAAMT1* gene, encoding the *M. truncatula* anthranilic acid methyltransferase 1, was introduced as a single-copy genomic integration into the strain 4, and in the parental strain. The *MtAAMT1* gene was expressed using the synthetic expression system (SES) [24] to achieve constitutive high production of the enzyme (see Additional file 1: Fig. S2). The identity of the products were confirmed by UPLC-MS analysis (see Additional file 1: Fig. S3). The modified strain 4 produced Me-ANTH to amounts similar to the amounts of ANTH produced by the original stain 4 (Fig. 4), indicating virtually complete conversion of ANTH into Me-ANTH by the introduced methyltransferase. The titer of Me-ANTH exceeded 400 mg l^−1^ in YPD medium at day 5.

**Fig. 5:**
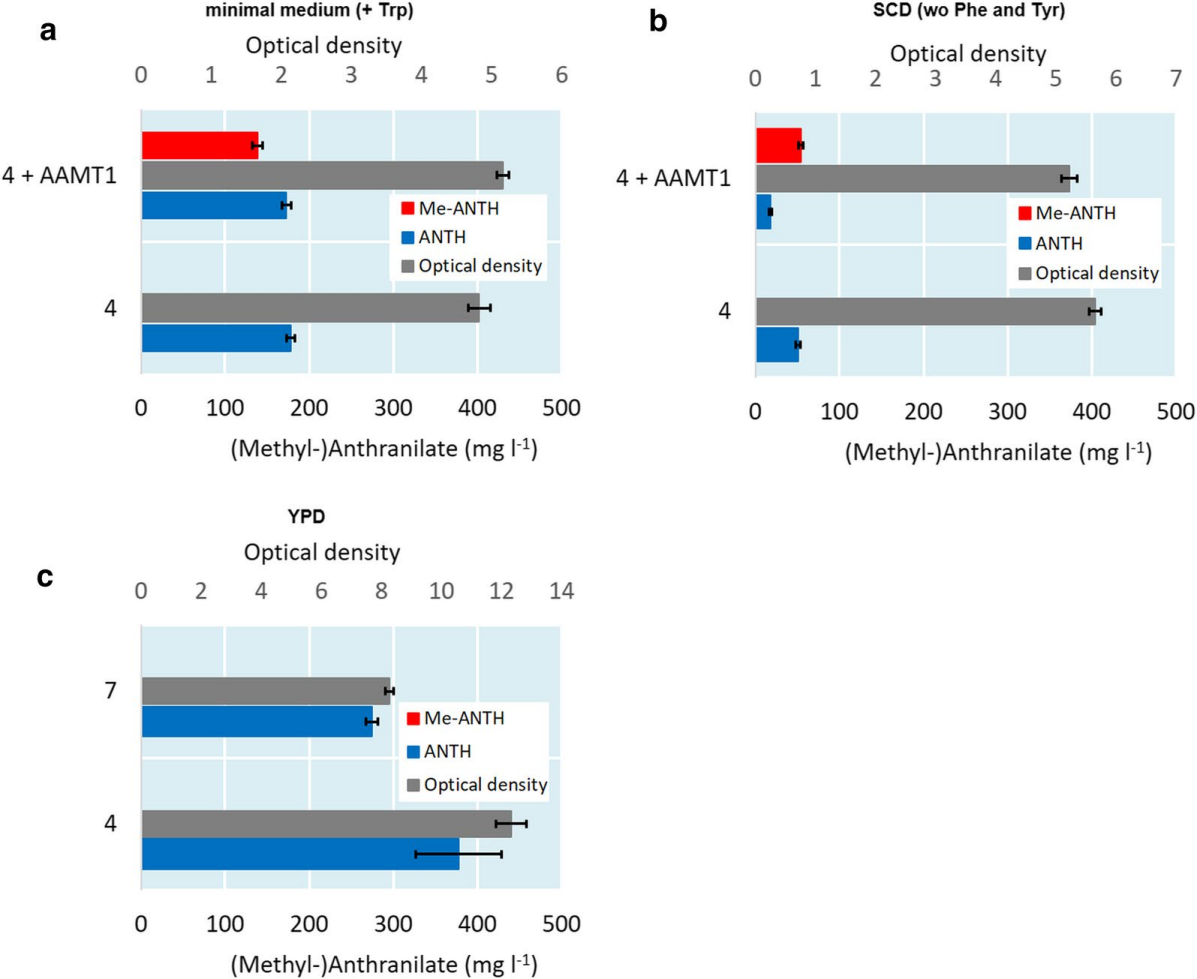
OD values and ANTH and Me-ANTH titers in the cultivations of strains 4, with or without *MtAAMT1* expression, and 7. The strains were cultivated for 5 days in minimal medium (**a**), SCD medium (**b**) or YPD medium (**c**) and the products were measured in the culture supernatants

In order to verify the suitability of the strain 4 as a chassis and YPD as a medium for Me-ANTH production, strains 4 and 7 were cultivated in YPD medium and strain 4, with or without *MtAAMT1* expression, in SCD medium or minimal medium for 5 days. OD values were measured and ANTH and Me-ANTH titers determined from samples taken at the end point of the cultivation (Fig. 5). Strain 4 grew to a higher OD and accumulated more ANTH than strain 7 in YPD medium (Fig. 5c), indicating that strain 4 is more suitable as a production host in this production medium. ANTH titers obtained with strain 4 and Me-ANTH titers obtained with strain 4 expressing *MtAAMT1* were much lower in SCD medium (Fig. 5b) and minimal medium (Fig. 5a) than in YPD medium (Figs. 4, 5c). Furthermore, conversion of ANTH to Me-ANTH by strain 4 expressing *MtAAMT1* was much less complete in SCD medium (Fig. 5b) and minimal medium (Fig. 5a) than in YPD medium (Fig. 4).

## Discussion

ANTH overproduction has been engineered in several prokaryotes but the suitability of eukaryotic species for ANTH production has received little attention. Only the patent application by Jäger et al. [14] suggest that ANTH production can be triggered in *S. cerevisiae* by disruption of *TRP4*. We attempted to engineer an efficient ANTH producing *S. cerevisiae* by first disrupting *TRP4* from the genome of *S. cerevisiae* CEN.PK113-1A and then carrying out extensive modifications to its genome to improve carbon flux towards ANTH biosynthesis. We introduced *ARO4*^*K229L*^, a gene for Aro4 mutant insensitive to feed-back inhibition by Tyr [25], to the *TRP4* locus. *ARO4*^*K229L*^ has previously been successfully used in *S. cerevisiae* for example to improve the production of CCM [26], and double the product yield without affecting growth in a *S. cerevisiae* strain engineered to produce coumarate [27]. Here, the use of *ARO4*^*K229L*^ improved ANTH titer in both media tested but did not affect growth in minimal medium and only very slightly in SCD medium.

We overexpressed *TRP2*^*S76L*^ and *TRP3* to funnel carbon towards ANTH production and reduce the flux to Phe and Tyr biosynthesis. This modification reduced OD slightly but did not affect ANTH titers significantly, thus increasing the OD normalized ANTH production. In the reaction catalyzed by Trp2 and Trp3 a pyruvate molecule is also formed. This pyruvate-forming reaction has been used to couple ANTH production to growth in *E. coli* by knocking out all other metabolic reactions producing pyruvate [28].

It has been shown that the overexpression of *ARO1* and *ARO2* improves Tyr production in rich medium in a metabolically engineered *S. cerevisiae* strain [29]. This strain, which had also *TKL1* expression upregulated, had also a slight growth defect [29]. In the experiments of Gold et al. [27], overexpression of *ARO1* in a *S. cerevisiae* strain deleted for *ARO10* knockout and expressing the *ARO4*^*K229L*^ mutant improved production of the heterologous product coumarate without a significant effects on growth in the minimal medium. We show here that the activation of *ARO1* and *ARO2* improves ANTH production and reduces growth of the engineered *S. cerevisiae* in minimal but not in SCD medium (strain 4 → 5). The *p*-hydroxyphenylpyruvate, an intermediate of Tyr biosynthesis, acts as an inhibitor to *S. cerevisiae* transketolase [30]. Thus, the overexpression of *ARO1* and *ARO2*, which increase flux towards Tyr biosynthesis, might have reduced transketolase activity. This would explain why overexpression of *TKL1* (strain 5 → 6) restored growth slightly in minimal medium. As a growth defect was not seen in the amino acid containing SCD medium, it is likely that the reduction in growth in the minimal medium was due to reduced synthesis of some other metabolites, such as E4P. Furthermore, downregulation of the gene for pyruvate kinase, *CDC19*, drastically improved the growth of the strain 5. Decrease in pyruvate kinase activity can be expected to result in the accumulation of three carbon intermediates of glycolysis, including G3P which is converted to E4P and X5P in a reaction that consumes also one molecule of F6P. Thus, it seems likely that the overexpression of *ARO1* and *ARO2* resulted in a growth defect through the depletion of the E4P pool, either directly by funnelling E4P towards SA pathway or by *p*-hydroxyphenylpyruvate-mediated transketolase inhibition. Deletion of glucose-6-phosphate dehydrogenase, *ZWF1*, and activation of transketolase, *TKL1*, have been shown to improve the product yield of the SA pathway derived CCM [26]. However, deletion of *ZWF* decreases the NADPH pool and if the product synthesis requires NADPH, the *ZWF1* deletion may have a negative effect as shown previously [31]. For this reason, we only activated the *TKL1* but did not delete *ZWF1* (strain 5 → 6).

Expression of Gln synthase (*glnA*) has been shown to increase ANTH yield in a minimal medium of a metabolically engineered *E. coli* strain [10]. Glu has been also shown to accumulate as a major side-product in an *E. coli* strain engineered for Trp production [32]. We upregulated *GLN1* expression in the engineered *S. cerevisiae* but did not observe improved ANTH titer in minimal medium while in SCD medium the ANTH titer improved significantly. It could be that either concentration of Gln is not limiting ANTH production or that the substrate of Gln synthase, Glu, is not present at sufficiently high concentration to affect ANTH production when strain 6 is cultivated in minimal medium.

Down-regulation of pyruvate kinase reaction has been considered as a potential approach to redirect the carbon flux towards aromatic amino acid biosynthesis. However, introduction of the mutation T21E in the *CDC19* gene, which is known to reduce pyruvate kinase activity, has been shown not to increase flux of carbon to the aromatic amino acid biosynthesis pathway [27]. As pointed out by Suástegui et al. [31], this experiment was carried out in a *ZWF1* knockout strain which might have affected the results. Since it has been shown that the *CDC19* null mutant cannot grow on glucose [33], we attempted to increase availability of PEP to the SA pathway in the strains 5–7 by downregulating pyruvate kinase activity. Growth was negatively affected in both SCD and minimal media for strains 6 and 7. In contrast, growth of strain 5 was greatly improved in minimal medium. ANTH titer improved for strain 7 in SCD medium and for strain 5 in minimal medium, while in other cases the ANTH titers either decreased or remained at the same level. Therefore, it seems that the effects of pyruvate kinase down-regulation are highly dependent on the genetic background of the strain.

ANTH production has been successfully engineered in several prokaryotic microorganisms. For example, *P. putida* has been engineered to produce a titer of 0.25 g l^−1^ of ANTH in a shake flask cultivation and a titer of 1.54 g l^−1^ and a yield of 0.035 g_ANTH_ g_glucose_^−1^ in a fed-batch cultivation in a minimal medium [5]. *E. coli* engineered for ANTH production produced a titer of 0.75 g l^−1^ and a yield of 0.20 g_ANTH_ g_glucose_^−1^ in a batch cultivation in a minimal medium and a titer of 14 g l^−1^ and a yield of 0.20 g_ANTH_ g_glucose_^−1^ in a fed-batch cultivation in rich medium [8]. *E. coli* and *C. glutamicum* have also been engineered for Me-ANTH production [9]. By fed-batch cultivating these strains in minimal medium, a Me-ANTH titer of 4.47 g l^−1^ and a yield of 0.045 g_Me-ANTH_ g_glucose_^−1^ were obtained with *E. coli* and a titer of 5.74 g l^−1^ and a yield of 0.020 g_Me-ANTH_ g_glucose_^−1^ were obtained with *C. glutamicum*. Unlike prokaryotic host organisms, such as *E. coli* and *P. putida* which lack internal membranes, *S. cerevisiae* can express cytochrome P450 oxidases of eukaryotic origin [12], allowing production of a broader range of ANTH derived metabolites. Furthermore, ANTH production in *S. cerevisiae* is uncoupled from growth [14], allowing larger portion of the carbon source to be used in ANTH production. Currently the only reported attempt to engineer an eukaryote to produce ANTH can be found in the patent application by Jäger et al. who claim to have produced 320 mg l^−1^ of ANTH using an *S. cerevisiae TRP4* knockout strain [14].

## Conclusions

Here, we first knocked out the *TRP4* gene from the genome of *S. cerevisiae* and then further engineered its metabolism for improved ANTH production. By carrying out batch cultivations with a strain optimized for ANTH production, we could obtain a titer of 199.7 mg l^−1^ in a minimal medium and a titer of 567.9 mg l^−1^ in SCD medium. Thus, we were able to nearly double the previously highest ANTH titer obtained with an eukaryotic microorganism. As these titers were obtained by cultivating the engineered *S. cerevisiae* strain on a microplate, drastically higher titers could be obtainable by controlled bioreactor cultivations *e.g.* in fed-batch mode. Furthermore, we show that these engineered *S. cerevisiae* strains are suitable for production of high value ANTH-derived compounds. By extending the pathway with a recently described anthranilic acid methyl transferase MtAAMT1 from *M. truncatula* [34] the industrially valuable flavor compound Me-ANTH accumulated. While the ANTH titers still remain relatively low compared to the values obtained with prokaryotes, we foresee that the strains presented here can serve as a basis for the development of efficient host organisms for the production of many ANTH-derived compounds.

## Methods

### Strains

All the plasmids were produced in *E. coli* TOP10 cells. The *S. cerevisiae* strains CEN.PK113-1A (*MATα URA3 HIS3 LEU2 TRP1 MAL2-8c SUC2*) and CEN.PK113-17A (*MATα ura3-52 HIS3 leu2-3,112 TRP1 MAL2-8c SUC2*) were used as parental strains. Detailed description of the engineered *S. cerevisiae* strains used in this study is shown in Fig. 1b. The strains used in the experiments where the Me-ANTH production was tested (shown in Figs. 4, 5) were constructed as follows. The parental strain and the strain 4 were transformed with the *MtAAMT1* expression cassette released from the pHIS3in-kanMX_SES-MtAAMT1 plasmid by *NotI*. The corresponding negative control strains were also constructed by transformations of the *kanMX* selection marker cassette released from the plasmid pHIS3in-kanMX by *NotI*. The transformed strains were selected for growth in presence of G-418 (YPD agar with 200 mg l^−1^ of G-418), and tested for the correct genomic integrations into the *HIS3* locus by qPCR (primers listed in Table 1).

**Table 1:** Primers used in the construction of the strains

Primer	Sequence (5′ → 3′)
oJKLiF-016	TGCTTAAATCTATAACTACAAAAAACACATACAGGAATTCCACCATGAGTGAATCTCCAATGTT
oJKLiF-017	CGTTACCCTTAGTAGTGGTGATAGCAGCAACACCATGCAAAGTAACACCCATGAAATGGTG
oJKLiF-018	AGCCGCTGCTCATTCTCACCATTTCATGGGTGTTACTTTGCATGGTGTTGCTGCTATCAC
oJKLiF-019	CTTATTCAGTTAGCTAGCTGAGCTCGACTCTAGAGGATCCCTATTTCTTGTTAACTTCTC
oJKLiF-084	ACTGACAACTAAGTGAATTTAAACTGCAATAATCACAAGAAAACTTAGTATTCCCTTATCCTAGTGTTTAAAGATTACGG
oJKLiF-085	TTCAGCTTAGTACAAATAAAACGTCTCTATCTAGTTTAAGTATAACATATGAAATAATGTCGAATTGGAGCTAGACAAAG
oJKLiF-133	GCATCGTCTCATCGGTCTCATATGACCGCTTCCATCAAAAT
oJKLiF-134	GTGGCGAGATACCTATGAATAAATAACGATCTAATTCATTA
oJKLiF-135	TATTCATAGGTATCTCGCCAC
oJKLiF-136	ATGCCGTCTCAGGTCTCAGGATTTAAGCTGATCCTACGATAT
oJKLiF-137	GCATCGTCTCATCGGTCTCATATGTCTGTGCACGCTGCAAC
oJKLiF-138	ATGCCGTCTCAGGTCTCAGGATTTATTCGCATAATTCATGAATG
oNNF-003	GACACCCGACAGATCAAGGC
oNNF-004	GCACGGTTATCCACAGAATC
8BS_mCh_GA_F	GATACTAACGCCGCCATCCAGTGTCGGCGCGCCGAATTAACCCTCACTAAAGG
8BS_mCh_GA_R	GATTCCATTGTAGATATTTAATTATGTGTGTTTATTCGAAACTAAGTTCTTG
sTF_noPacI_GA_F	CGAATAAACACACATAATTAAATATCTACAATGGAATCTACTCCTACTAAGCAAAAAGC
sTF_noPacI_GA_R	GCTGCAGCTTTAAATAATCGGTGTCAGATATCAGGATCAATGGCGTCGGACA
MtAAMT1_GA_F	CTGCTTATCAACACACAAACACTTAATTAAAATGGAAGTTGCTCAAGTTTTACCAATG
MtAAMT1_GA_R	CATAAATCATAAGAAATTCGGATCCTCAAGCTTTTCTAGTCATCATTAAAGTCAAATTAG
MtAAMT1_qPCR_F	GAAATGGAAACTGAAATGGGTC
MtAAMT1_qPCR_R	CGTTAGATGGTGATGTAGAAGTC
Sc_HIS3_qPCR_F	TCGCAAGTGATTAACGTCCA
Sc_HIS3_qPCR_R	CGCAAATCCTGATCCAAACCT
kanMX_qPCR_F	TATTGTTGATGCGCTGGCAG
kanMX_qPCR_R	TATTCATTCGTGATTGCGCC

### Media and culture conditions

Luria Bertani Broth culture medium supplemented with 100 µg ml^−1^ of ampicillin or 25 µg ml^−1^ of chloramphenicol and culture conditions of 37 °C and 250 rpm were used with *E. coli*. YPD medium (10 g l^−1^ yeast extract, 20 g l^−1^ peptone and 20 g l^−1^ D-glucose) with 200 µg ml^−1^ of G418 was used for *S. cerevisiae* pre-cultures. For selection of transformants with *TRP2*^*S76L*^ and *TRP3* integration cassettes, SCD-Ura-Leu (uracil and leucine deficient synthetic complete media supplemented with 20 g D-glucose l^−1^) plates were used for uracil auxotrophic selection. All the *S. cerevisiae* cultivations were carried out at 30 °C and small scale liquid cultivations were shaken at 250 rpm.

The characterization of the engineered *S. cerevisiae* strains was carried out in 96-well plates using a Beckman Coulter screening and cultivation robot as described previously [18]. Briefly, two sequential 24-h pre-cultivation steps in YPD and SCD-Phe-Tyr (Phe and Tyr deficient synthetic complete media supplemented with 20 g l^−1^ D-glucose) or defined minimal medium [35] (supplemented with 0.4 mM Trp and 10 g l^−1^ D-glucose), respectively, were carried out (Fig. 1c). The final cultivations were done in SCD-Phe-Tyr medium for 45 h and minimal medium for 72 h. The cultivation volume of 150 µl was used in each step and inoculation volume of 10 µl in each transfer. Cultivations were monitored by measuring OD at 595 nm in 2-h intervals. The strains were further characterized by cultivating 5 days in 50 ml of SCD-Phe-Tyr medium or minimal medium (strain 4) or YPD medium (strains 4 and 7). OD was measured and UPLC-MS samples taken at the end point of the cultivation.

The production of Me-ANTH was performed in 50 ml of SCD-Phe-Tyr medium, minimal medium or YPD medium. The strains were cultivated in duplicate (YPD medium) or triplicate (SCD-Phe-Tyr medium and minimal medium) liquid cultures in 250 ml Erlenmeyer flasks for 5 days at 30 °C with a shaking of 220 rpm. The culture supernatant samples were collected by centrifugation in day 2 and day 5, and subjected to the UPLC-MS analysis.

Unless stated otherwise, all cultivations were carried out in triplicates. Figures 2, 3, 4, 5 represent average values from measurements taken from these cultivations and error bars represent standard deviations.

### Plasmids and DNA construction

Expression cassette for the allosterically insensitive DAHP synthase *ARO4*^*K229L*^ was constructed by PCR amplifying the *ARO4*^*K229L*^ open reading frame (ORF) in two parts from *S. cerevisiae* genomic DNA with the primers oJKLiF-016/-017 and oJKLiF-018/-019 (Table 1). The primers oJKLiF-017 and -018 introduced the *K229L* mutation and contained the homology arms for mutual recombination. Primers oJKLiF-016 and -019 contained the homology arms for the *EcoRI* and *BamHI* digested yeast expression plasmid derived from pYX212 [36]. These two PCR fragments were combined and cloned into the digested expression plasmid backbone using the yeast homologous recombination. As a result, *ARO4*^*K229L*^ was in the yeast expression plasmid under *TPI1p*. For genomic integrations, *ARO4*^*K229*^ expression cassette was PCR amplified with the primers oJKLiF-084/-085, introducing homology arms for the genomic *TRP4* integration, respectively. Similar to *ARO4*^*K229L*^, allosterically insensitive *TRP2*^*S76L*^ mutant was PCR amplified in two fragments with the primers oJKLiF-133/-134 and oJKLiF-135/-136. The resulting fragments were fused with an additional PCR using primers oJKLiF-133/-136 and transferred to Yeast MoClo entry vector [23]. *TRP3* gene was PCR amplified with the primers oJKLiF-137/-138 and transferred to MoClo entry vector. Yeast MoClo method was used to construct integration cassettes for *TRP2*^*S76L*^ and *TRP3*. *TRP2*^*S76L*^ cassette composed of the following building blocks: pYTK-002, -009, -051, -067, -074, -087, -089 and -093 resulting in *LEU2* targeted integration cassette with *URA3* selection marker and *TRP2*^*S76L*^ expressed under *TDH3p*. The corresponding composition of the *TRP3* cassette was: pYTK-003, -011, -052, -068, -075, -086, -089 and -092 resulting in *URA3* targeted integration cassette with *LEU2* selection marker and *TRP3* expressed under *PGK1p*. Promoter switches for the genes *ARO1*, *ARO2*, *GLN1* and *CDC19* were carried out by using PCR amplified promoters flanked with homology arms as a donor DNA.

The expression cassette for the *MtAAMT1* was constructed in the *S. cerevisiae* integrative plasmid targeting the *HIS3* locus. The plasmid was based on previously constructed pHIS3in-SpHIS5 and pGRE3in-kanMX plasmids [37]. The *kanMX* selection marker from the pGRE3in-kanMX was cloned in place of the *SpHIS5* marker in the pHIS3in-SpHIS5 between *AscI* and *BglII* restrictions sites to make the pHIS3in-kanMX plasmid. This plasmid was further modified by inserting DNA parts from plasmid pLEU2in-SES-A* [24]. Two DNA fragments from the pLEU2in-SES-A* were obtained by PCR with primers 8BS_mCh_GA_F and 8BS_mCh_GA_R and sTF_noPacI_GA_F and sTF_noPacI_GA_R (Table 1), and these two fragment were assembled between *AscI* and *EcoRI* of the pHIS3in-kanMX by the Gibson assembly method (NEB). The resulting plasmid pHIS3in-kanMX_SES-A* was used for the final construction step, where the PCR product containing the *S. cerevisiae* codon-optimized MtAAMT1 coding region (*MtAAMT1* from *Medicago truncatula*; NCBI Reference Sequence: XP_003604791.1; codon optimized and ordered as a synthetic gene (Genescript, USA) with suitable DNA flanks) was inserted between *PacI* and *BamHI* sites to form pHIS3in-kanMX_SES-MtAAMT1. The MtAAMT1 coding sequence was amplified by PCR using primers MtAAMT1_GA_F and MtAAMT1_GA_R (Table 1). The final expression cassette was confirmed by sequencing.

### *S. cerevisiae* genome editing

For the CRISPR/Cas9 mediated genomic integrations, an approach based on a two plasmid system–*Cas9* and single-guide RNA (sgRNA) expressed from separate plasmids–was used similar as described previously [18]. The Cas9 expression vector was constructed from Yeast MoClo parts pYTK-002, -013, -036, -051, -067, -077, -081 and -083 resulting in a *CEN/ARS* plasmid with *KanR* selection marker and *Cas9* expressed under *TEF1p*. All the sgRNA plasmids were based on a backbone which was constructed from the Yeast MoClo parts pYTK-002, -013, -051, -067, -078, -082 and -083 resulting in a 2µ plasmid with *NatR* selection marker. This backbone was PCR amplified with the primers oNNF-003/-004 and was co-transformed into *S. cerevisiae* together with a PCR amplified DNA fragment containing an expression cassette for sgRNAs (expressed between *pSNR52* and *tSUP4*). The workflow of the CRISPR/Cas9 method is described in the Additional file 1: Fig. S1 and the oligos for construction of each sgRNA expression cassettes are listed in Table 1. All *S. cerevisiae* transformations were carried out by using the Gietz transformation method [38]. About 0.5–1 µg of sgRNA plasmid backbone and expression cassette and 1–5 µg of donor DNA was used in the transformations.

### Chemical analyses

The yeast liquid cultures were centrifuged and ANTH / Me-ANTH titers were analyzed from the supernatants. UPLC was carried out using ACQUITY UPLC BEH HSS T3 Column, 1.8 µm, 2.1 mm X 100 mm (Waters) kept at 45 °C. An injection volume of 2 µl was used. Flow rate of the mobile phase A (0.1% of formic acid in water) and B (0.1% of formic acid in methanol) was 0.4 ml min^−1^. Following gradient program was used: 5.0% of B at 0.0 min, 60.9% of B at 4.3 min, 97.1% of B at 5.6 min, and 95.0% of B at 6.0 min. Equilibrium time of 2.0 min was used between runs.

Mass spectrometry was carried out using electrospray ionization (ESI) in positive polarity. The capillary voltage was 3.0 kV, cone voltage 30 kV, source temperature 150 ºC and desolvation temperature 500 ºC. The cone and desolvation gas flow were set at 150 l h^−1^ (nitrogen) and 1000 l h^−1^ (nitrogen), respectively, collision gas was 0.15 ml min^−1^. The analysis was performed with ANTH and Me-ANTH as analytical standards for the identification and quantification of the products.

## Supplementary Information

**Additional file 1: Fig. S1** The workflow of the CRISPR/Cas9 method used in the strain construction. The *S. cerevisiae* strain was first transformed with the Cas9 expression vector and then with the linear fragments of the sgRNA expression cassette, the sgRNA expression plasmid backbone and the donor DNA. The sgRNA expression cassettes were produced by two PCR reactions. The first PCR produces the target specific 20 nucleotide sequence and results in two fragments. The second PCR then amplifies the whole cassette fusing these fragments together. The sgRNA expression plasmid is formed by homologous recombination in vivo from the sgRNA expression cassette and the plasmid backbone. After each round of genomic integrations, the sgRNA expression plasmid is dropped from the cells by growing on non-selective medium which allows new round of modification to be carried out. **Fig. S2** The expression cassette used for the *MtAAMT1* expression in the *S. cerevisiae* strain engineered for ANTH production (strain 4). **Fig. S3** Example of UPLC-MS analysis of ANTH and Me-ANTH. The top panel represents the base peak intensity (BPI) chromatograms of analytical standards, ANTH and Me-ANTH as detected by mass spectrometry. The lower panels show the same analysis of production of the compounds by the strain 4 and the strain 4 modified by the expression cassette for *MtAAMT1.*

## Data Availability

The datasets used and/or analysed during the current study are available from the corresponding author on reasonable request.

## Abbreviations

ANTH: Anthranilate
CCM: Cis,cis-muconic acid
CHO: Chorismate
DAHP: 3-Deoxy-D-arabino-heptulosonate-7-phosphate
DHQ: 3-Dehydroquinate
DHS: 3-Dehydroshikimate
E4P: Erythrose-4-phosphate
EPSP: 5-Enolpyruvyl-shikimate-3-phosphate
ESI: Electrospray ionization
F6P: Fructose-6-phosphate
G3P: Glyceraldehyde-3-phosphate
GLC: Glucose
Glu: Glutamate
Gln: Glutamine
Me-ANTH: Methyl anthranilate
MtAAMT1: *Medicago truncatula* Anthranilic acid methyltransferase 1
PEP: Phosphoenolpyruvate
Phe: Phenylalanine
PPP: Pentose phosphate pathway
PYR: Pyruvate
S3P: Shikimate-3-phosphate
SA: Shikimate
SES: Synthetic expression system
Trp: Tryptophan
Tyr: Tyrosine
X5P: Xylulose-5-phosphate
