# Supplementary material for: Engineering of Saccharomyces cerevisiae for anthranilate and methyl anthranilate production
Source: Microb Cell Fact. 2021 Feb 3;20:34. doi: 10.1186/s12934-021-01532-3 (PMC7860014; doi:10.1186/s12934-021-01532-3)
Supplement: Supplementary file 1 — Additional file 1: Fig. S1 The workflow of the CRISPR/Cas9 method used in the strain construction. The S. cerevisiae strain was first transformed with the Cas9 expression vector and then with the linear fragments of the sgRNA expression cassette, the sgRNA expression plasmid backbone and the donor DNA. The sgRNA expression cassettes were produced by two PCR reactions. The first PCR produces the target specific 20 nucleotide sequence and results in two fragments. The second PCR then amplifies the whole cassette fusing these fragments together. The sgRNA expression plasmid is formed by homologous recombination in vivo from the sgRNA expression cassette and the plasmid backbone. After each round of genomic integrations, the sgRNA expression plasmid is dropped from the cells by growing on non-selective medium which allows new round of modification to be carried out. Fig. S2 The expression cassette used for the MtAAMT1 expression in the S. cerevisiae strain engineered for ANTH production (strain 4). Fig. S3 Example of UPLC-MS analysis of ANTH and Me-ANTH. The top panel represents the base peak intensity (BPI) chromatograms of analytical standards, ANTH and Me-ANTH as detected by mass spectrometry. The lower panels show the same analysis of production of the compounds by the strain 4 and the strain 4 modified by the expression cassette for MtAAMT1. [file 12934_2021_1532_MOESM1_ESM.docx]

Additional file 1

# Engineering of *Saccharomyces cerevisiae* for anthranilate and methyl anthranilate production

Joosu Kuivanen^1,2^, Matti Kannisto^1*^, Dominik Mojzita^1^, Heiko Rischer^1^, Mervi Toivari^1^, Jussi Jäntti^1^

^*^Correspondence: Matti.Kannisto@vtt.fi.

^1^VTT Technical Research Centre of Finland Ltd, Espoo, Finland.

^2^Present address: eniferBio Oy, Espoo, Finland.


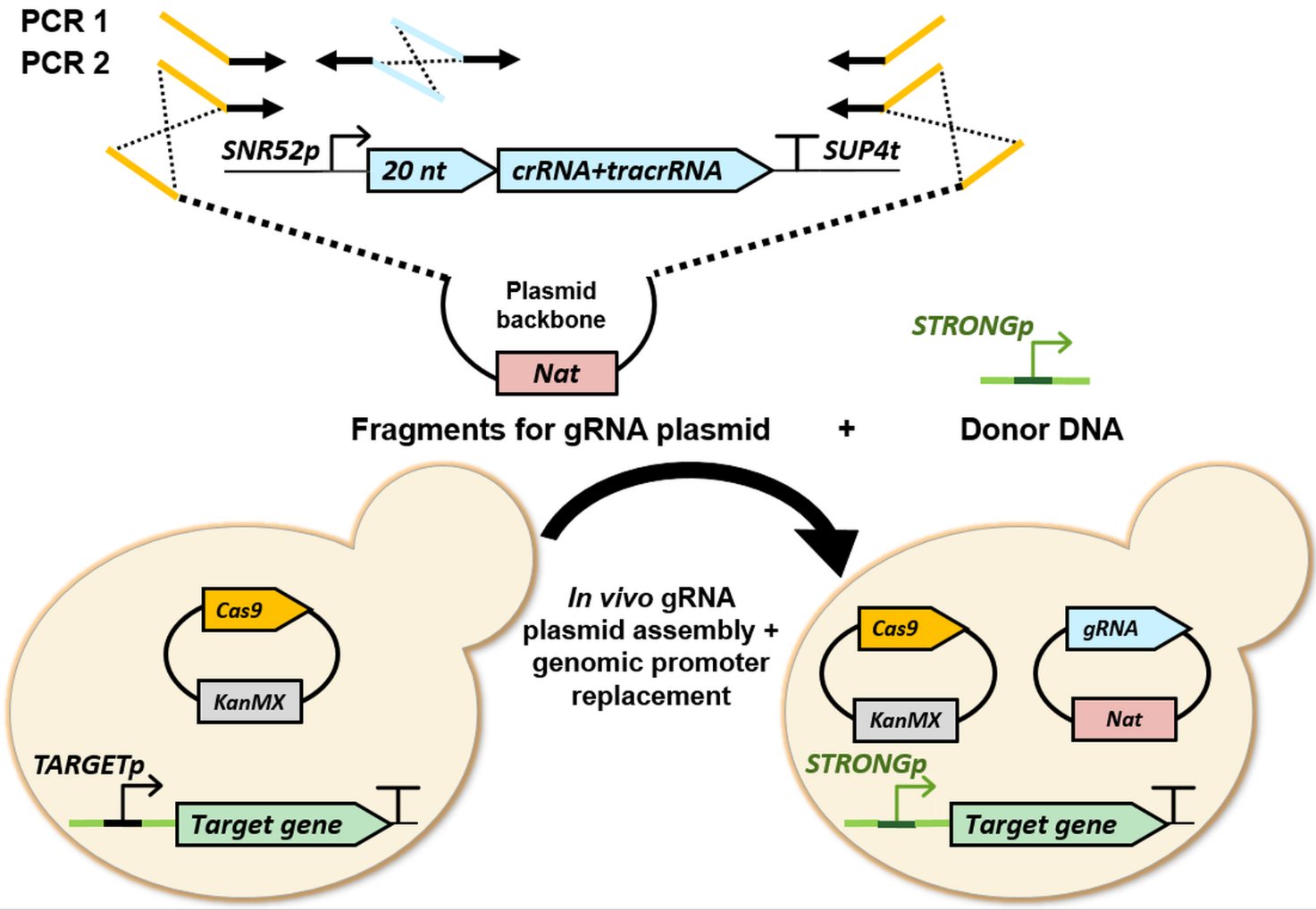


**Fig. S1** The workflow of the CRISPR/Cas9 method used in the strain construction. The *S. cerevisiae* strain was first transformed with the Cas9 expression vector and then with the linear fragments of the sgRNA expression cassette, the sgRNA expression plasmid backbone and the donor DNA. The sgRNA expression cassettes were produced by two PCR reactions. The first PCR produces the target specific 20 nucleotide sequence and results in two fragments. The second PCR then amplifies the whole cassette fusing these fragments together. The sgRNA expression plasmid is formed by homologous recombination *in vivo* from the sgRNA expression cassette and the plasmid backbone. After each round of genomic integrations, the sgRNA expression plasmid is dropped from the cells by growing on non-selective medium which allows new round of modification to be carried out


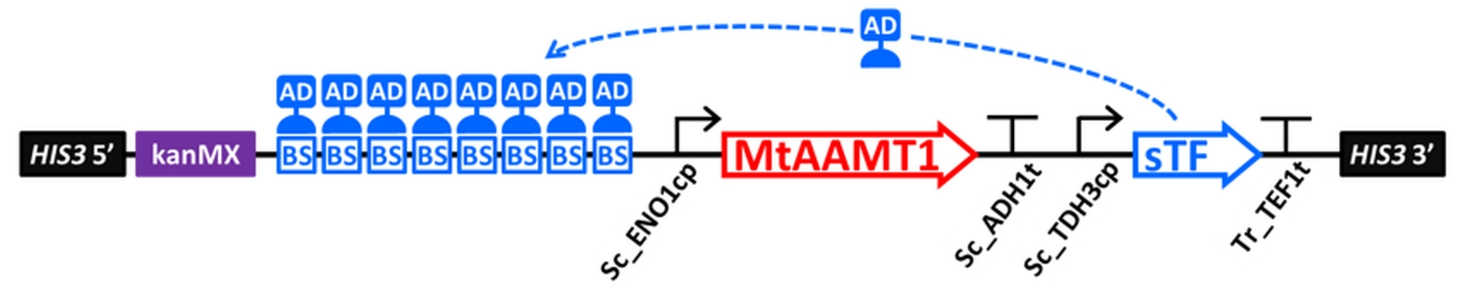


**Fig. S2** The expression cassette used for the *MtAAMT1* expression in the *S. cerevisiae* strain engineered for ANTH production (strain 4)


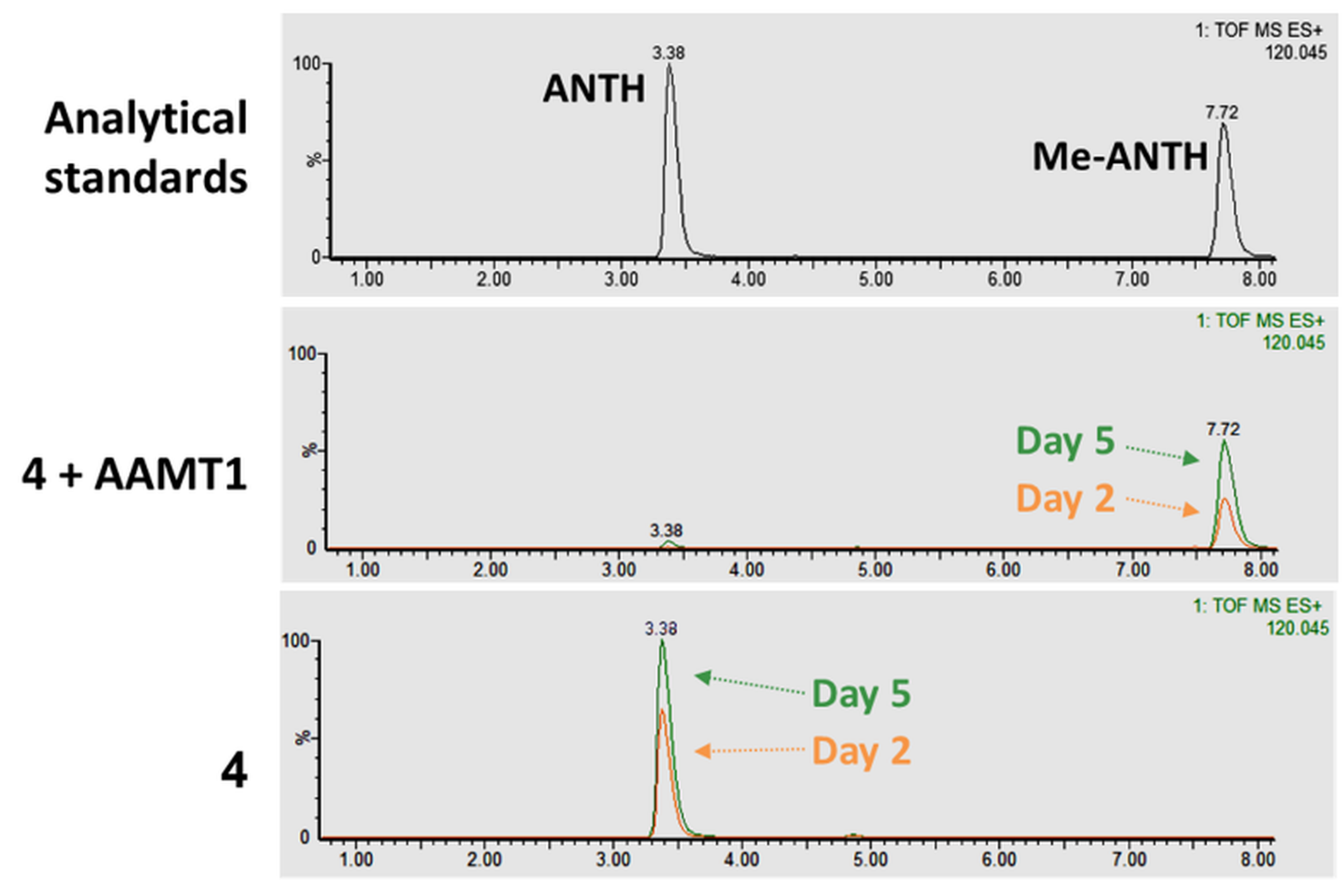


**Fig. S3** Example of UPLC-MS analysis of ANTH and Me-ANTH. The top panel represents the base peak intensity (BPI) chromatograms of analytical standards, ANTH and Me-ANTH as detected by mass spectrometry. The lower panels show the same analysis of production of the compounds by the strain 4 and the strain 4 modified by the expression cassette for *MtAAMT1*
